# Systematic pan-cancer analysis identifies RALA as a tumor targeting immune therapeutic and prognostic marker

**DOI:** 10.3389/fimmu.2022.1046044

**Published:** 2022-11-17

**Authors:** Haoer Jin, Sha Qin, Jiang He, Juxiong Xiao, Qingling Li, Yitao Mao, Luqing Zhao

**Affiliations:** ^1^ Department of Pathology, Xiangya Hospital, Central South University, Changsha, Hunan, China; ^2^ Department of Pathology, School of Basic Medical Science, Xiangya School of Medicine, Central South University, Changsha, Hunan, China; ^3^ Center for Molecular Medicine, Xiangya Hospital, Central South University, Changsha, Hunan, China; ^4^ Department of Radiology, Xiangya Hospital, Central South University, Changsha, Hunan, China; ^5^ National Clinical Research Center for Geriatric Disorders, Xiangya Hospital, Central South University, Changsha, Hunan, China

**Keywords:** RALA, pan-cancer analysis, HCC, immune target, prognostic marker

## Abstract

**Introduction:**

RALA is a member of the small GTPase Ras superfamily and has been shown to play a role in promoting cell proliferation and migration in most tumors, and increase the resistance of anticancer drugs such as imatinib and cisplatin. Although many literatures have studied the cancer-promoting mechanism of RALA, there is a lack of relevant pan-cancer analysis.

**Methods:**

This study systematically analyzed the differential expression and mutation of RALA in pan-cancer, including different tissues and cancer cell lines, and studied the prognosis and immune infiltration associated with RALA in various cancers. Next, based on the genes co-expressed with RALA in pan-cancer, we selected 241 genes with high correlation for enrichment analysis. In terms of pan-cancer, we also analyzed the protein-protein interaction pathway of RALA and the application of small molecule drug Guanosine-5'-Diphosphate. We screened hepatocellular cancer (HCC) to further study RALA.

**Results:**

The results indicated that RALA was highly expressed in most cancers. RALA was significantly correlated with the infiltration of B cells and macrophages, as well as the expression of immune checkpoint molecules such as CD274, CTLA4, HAVCR2 and LAG3, suggesting that RALA can be used as a kind of new pan-cancer immune marker. The main functions of 241 genes are mitosis and protein localization to nucleosome, which are related to cell cycle. For HCC, the results displayed that RALA was positively correlated with common intracellular signaling pathways such as angiogenesis and apoptosis.

**Discussion:**

In summary, RALA was closely related to the clinical prognosis and immune infiltration of various tumors, and RALA was expected to become a broad-spectrum molecular immune therapeutic target and prognostic marker for pan-cancer.

## Background

Cancer is now the leading cause of death in China and most developed countries. According to GLOBOCAN 2020, around 1930 million new cancer cases and 10 million cancer deaths worldwide in 2020 (1). Over the past decade, accelerated progress has been made in the design, improvement and application of anticancer therapies. Immunotherapy has become a new hot spot in clinical treatment of cancer. In particular, the application of a variety of immune checkpoint inhibitors in clinical practice has opened new direction for cancer treatment. Among them, cytotoxic T lymphocyte-associated antigen 4 (CTLA-4)/B7 and programmed cell death 1 (PD-1)/programmed cell death ligand 1 (PD-L1) are the most representative (2). But more and more clinical studies have shown that the use of checkpoint inhibitors can lead to multiple immune-related adverse events (irAEs) in cancer patients. The development of irAEs is related to irreversible organ damage, which may bring fatal risks. Organ damage in the endocrine system is most common, which mainly results in permanent damage to endocrine organs and usually requires long-term treatment (3–5). In order to increase efficacy and reduce side effects, new treatment-gene therapies came into being. Compared with other treatments, the advantage of gene therapy is that it can directly repair or even substitute pathogenic genes at the molecular level, and further rectify abnormal gene expression, so as to achieve precise and individualized treatment of cancer (6, 7). Therefore, the most principal objective is to screen out genes closely related to tumor growth and metastasis.

RAL (RAS-like) GTPases are encoded by RALA and RALB, which are located on human chromosomes 7 and 2, respectively. The proteins encoded by them have the same structural organization and the sequence identity is approximately 85% (8). RAS-dependent or RAS-independent upstream signals can activate RAL. In mammals, there are generally three downstream pathways of RAS signal, namely, RAF, PI3K and RAL-GEFs family (9, 10). RAL plays an important physiological role in normal cells. RAL-GEF/RAL signaling pathway can activate transcription factor STAT3 and JNK kinases by activating tyrosine kinase Src. The signal then activates c-jun kinase, which can stimulate transcription activity, cell differentiation and apoptosis (11). In the immune system, studies have shown that both RALA and RALB play key roles in immune response through cell-mediated cytotoxicity in natural killer (NK) cells (12). It was discovered more than 20 years ago that researchers had discovered the carcinogenic effect of RAL (13). In recent years, with the deepening research on cancer, molecule and immunology, the research direction of RAL function in cancer had been greatly expanded. Studies have found that RALA can enhance the proliferation, self-renewal and metastasis of HCC cells. The high expression of RALA in HCC is related to the increase of copy number, and is regulated and driven by the co-transcription of SP1 and ETS2. In addition, RALGAPA2 and RAL negative regulator, were down-regulated in HCC (14). However, the specific mechanism of RALA participating in pan-cancer immunity and progression remains unclear. In addition, the relationship between RALA level and various immune cell infiltration in tumor microenvironment (TME) has not been fully studied.

Thus, we studied the expression of RALA in TME and its correlation with immune infiltration and survival time of various cancers. Data showed that RALA had a cancer-promoting effect in most tumors, and increasing the expression level of RALA may reduce the lifetime of cancer patients. According to analysis, we found that the differential expression, prognosis and immune cell infiltration of RALA in HCC were statistically significant, and our team had a deep foundation in the study of RALA and HCC (14). Therefore, HCC was selected for further discussion and molecular biology verification to confirm the carcinogenic effect of RALA. Taken together, RALA is a promising therapeutic target for cancer and may serve as a marker of immune infiltration and poor prognosis.

## Materials and methods

### Data collection and differential expression analysis

Download RNA-seq, somatic mutation and related clinical data for 33 cancers from the Cancer Genome Atlas (TCGA) dataset (https://portal.gdc.cancer.gov/). Normal samples were selected from Genotype-Tissue Expression (GETx) dataset. The combined queue for TCGA and GTEx samples was downloaded from https://xenabrowser.net/, where batch effects had been removed. Cell line data were downloaded from the Human Protein Atlas (HPA) dataset. Correlation between gene expression and immune infiltration abundance in TIMER.

### Immunohistochemistry staining and immunofluorescence

In order to evaluate the difference in RALA expression at protein level, IHC images of RALA protein in normal tissues and four tumor tissues, including colorectal cancer, breast cancer, prostate cancer and lung cancer. Cellular localization of RALA by immunofluorescence. The data was downloaded and analyzed from HPA (http://www.proteinatlas.org/).

### Correlation of RALA expression with TMB and MSI

Tumor mutation burden (TMB) and microsatellite instability (MSI) are downloaded from TCGA database. TMB is usually defined as the total number of mutations in tumor samples, which is a promising biomarker for immune response (15). TMB is closely related to immune checkpoint inhibitors (ICIs) and was initially identified as a biomarker of ICIs in melanoma (16). In addition to TMB, MSI can also be used as a potential biomarker for predicting ICIs response. MSI is determined by calculating the total number of mutations per million base pairs. MSI activates anti-tumor immune response, leading to the accumulation of mutations and the formation of new antigens (17). Corresponding clinical information and RNA-sequencing expression profiles for RALA were downloaded from the TCGA dataset. TMB is derived from the article Thorsson V et al. gave a more detailed description of TMB in The Immune Landscape of Cancer (18), while Bonneville R et al. explained MSI in their article (19). We analyze all data using R version 4.0.3 and the corresponding R package. If not stated otherwise, two-group data performed by wilcox test. P values less than 0.05 were considered statistically significant.

### Functional enrichment analysis of RALA

Use the cBioPortal dataset to find genes co-expressed with RALA and select some genes with significant indigenous relevance (20). In order to further confirm the potential function of RALA, the data is analyzed through functional enrichment. Gene Ontology (GO) is a widely used tool for annotating functional genes, especially molecular function (MF), biological pathway (BP) and cellular component (CC). KEGG enrichment analysis is practical for analyzing gene function and related high-level genome function information. In order to better understand the carcinogenic effect of target genes, the ClusterProfiler package in R was used to analyze the GO function of potential mRNAs and enrich the KEGG pathway.

### Protein-protein interaction

String database (https://string-db.org/) is one of the most abundant and widely used databases for studying protein interactions (21). Studying the interaction network between proteins helps to mine the core regulatory genes. In our study, the String, PINA, BioGRID database were used to retrieve interactions between known and predicted proteins.

### RALA and pathways

Main pathway data containing RALA comes from the SMPDB database (https://smpdb.ca/) (22, 23). The correlation between RALA and each pathway was predicted in HCC, and analyzed by R package GSVA. The parameter method = ‘ssgsea’ was selected, and finally the correlation between gene and pathway score was analyzed by Spearman correlation (24, 25).

### Cell culture

PLC/PRF/5, Huh-7 and THLE-3 cells were purchased from Zhong Qiao Xin Zhou Biotechnology Co., Ltd. (Shanghai, China). All cell lines were cultured in MEM medium (Gibco, USA) containing 10% fetal bovine serum (FBS) (Gibco, USA), 100 μg/mL streptomycin and 100 IU/mL penicillin (Gibco, USA). They were cultured at 37°C, 5% CO2.

### Reverse transcription-quantitative polymerase chain reaction

Total RNA was extracted from HCC cell lines according to the RNA isolator (Vazyme, China), and the concentration and purity of total RNA were determined. The cDNA is obtained by reverse transcription of total RNA using the SureScript First-strand cDNA Synthesis Kit (Gene Copoeia, USA). RT-PCR assay was performed by Power SYBR Green (Takara, Hangzhou, Zhejiang, China). The relative expression of genes was calculated and standardized by 2-ΔΔCt method relative to β-actin. Specific primer sequences were as follows: RALA: 5’-ATGGCTGCAAATAAGCCCAAG-3’(forward), 5’-TGTCTGCTTTGGTAGGCTCATA-3’(reverse); β-actin: 5’- CATGTACGTTGCTATCCAGGC-3 (forward), 5′- CTCCTTAATGTCACGCACGAT-3’(reverse).

### Western blotting analysis

Whole cell lysates were collected by using RIPA lysates (Beyotime, China) and total protein concentrations were determined by BSA standard protein. The antibodies used in this study were RALA (ZENBIO, China) and β-actin (#20536-1-AP, Proteintech) antibodies. HRP-labeled anti-rabbit secondary antibody was used for ECL detection.

### Statistical analysis

RNA-seq data was converted into TPM (transcripts per 100 000 reads) format and log2 converted. Wilcoxon rank sum test was performed on these tumor types; p<0.05 was considered to indicate the differential expression between normal and tumor tissue. R software (version 4.0.3) was used for analysis, and the R package “ggplot2” was used to visualize the data and draw the box diagram.

## Results

### Differential expression of RALA in pan-cancer

**Figure 1:**
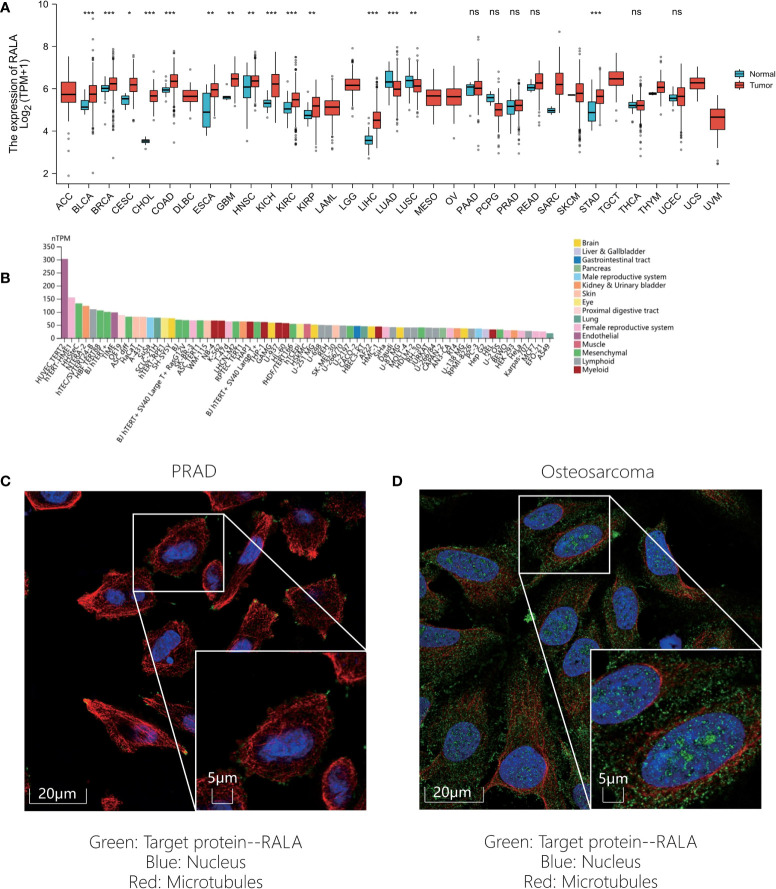
RALA expression level of pan-cancer in different databases. **(A)** RALA expression analyzed by TCGA dataset. **(B)** RNA expression data as normalized transcript per million (nTPM) values of tissue culture cell lines from HPA. **(C-D)** IF results for RALA expression levels in cancer. **(C)** PRAD. **(D)** Osteosarcoma. ns, no significance, *p < 0.05, **p < 0.01, ***p < 0.001.

In this paper, the expression of RALA in 33 kinds of cancers was compared based on TCGA database (**Figure 1A**). However, we found that adrenocortical cancer (ACC), lymphoid neoplasm diffuse large B-cell lymphoma (DLBC), acute myeloid leukemia (LAML), low grade glioma (LGG), mesothelioma (MESO), ovarian serous cystadenocarcinoma (OV), testicular cancer (TGCT), uterine carcinosarcoma (UCS), ocular melanomas (UVM) lack of gene expression data in normal tissues, so GTEx dataset was introduced and analyzed (**Supplementary Figures 1A, B**). In normal tissues, the five tissues with the highest expression level of RALA were lung, vagina, nerve, cervix uteri and skin (**Supplementary Figure 1B**). Except for LAML, lung adenocarcinoma (LUAD) and pheochromocytoma and paraganglioma (PCPG), RALA was higher in almost all cancer samples than in normal controls (**Supplementary Figure 1A**; P<0.001). RNA expression data as normalized transcript per million (nTPM) values of tissue culture cell lines. The analyzed cell lines are divided into 16 color-coded groups according to the organ they were obtained from (**Figure 1B**). The top three cancer cell lines with the highest nTPM values were NTERA-2, OE19 and A-431. NTERA-2 comes from the kidney and urinary bladder, OE19 mainly exists in the proximal digestive tract, namely esophagus; A-431 belongs to skin tissue.

**Figure 2:**
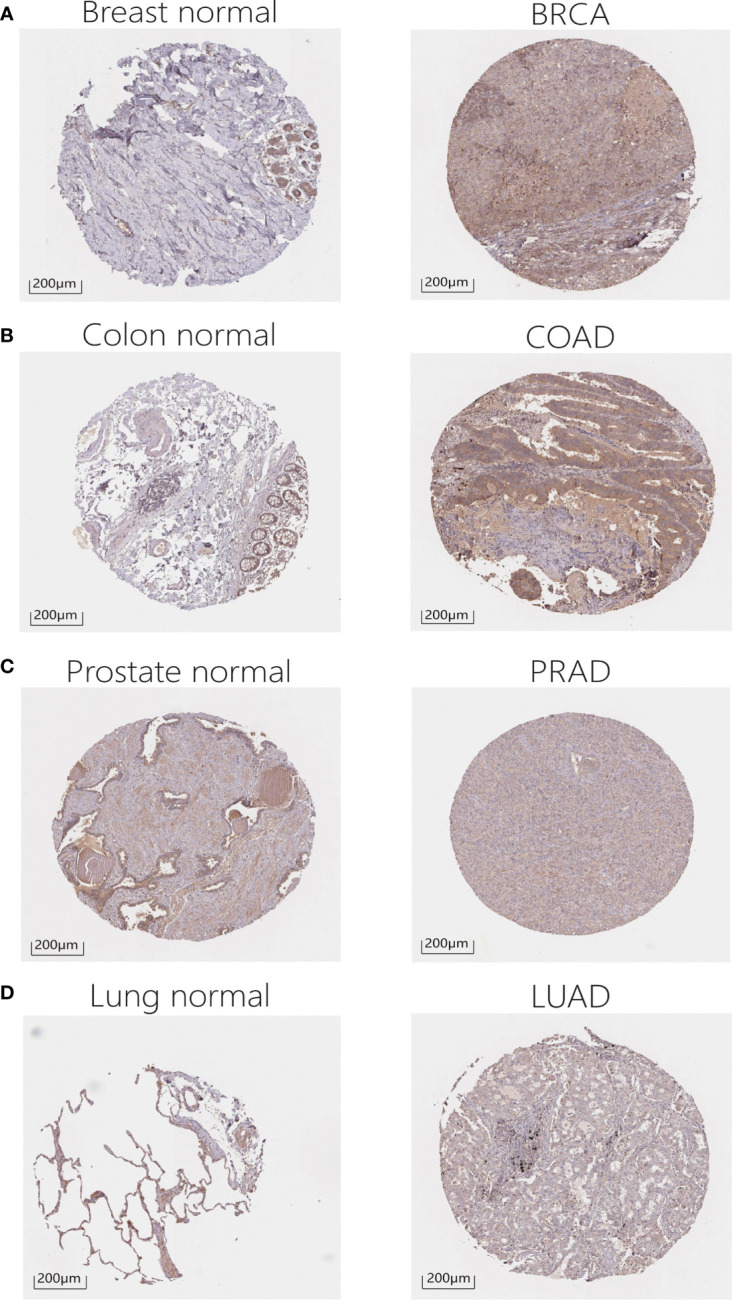
IHC results for RALA expression levels in cancers and normal tissues. **(A)** Breast. **(B)** Colon. **(C)** Prostate. **(D)** Lung.

In addition, in order to evaluate the protein level of RALA expression, we analyzed the IHC and IF results provided by HPA database and compared the results with the RALA gene expression data from TCGA. As shown in the diagram, the data analysis results of these two databases are consistent. Normal colon and breast tissues had mild or moderate RALA IHC staining, while tumor tissues had strong staining. On the contrary, the RALA staining of normal lung tissue samples was strong, while the LUSC and LUAD were weakly or moderately stained. The staining intensity in normal prostate tissue was similar to that in PRAD, and the expression level of RALA was not statistically different (**Figures 2A–D**). IF images include two cell lines, PC-3 and U-2OS, from PRAD and osteosarcoma, respectively. In osteosarcoma cells, RALA is mainly localized in the plasma membrane. In PRAD cells, in addition to the plasma membrane, some parts are confined to focal adhesion sites (**Figures 1C, D**).

### The prognostic value of RALA

**Figure 3:**
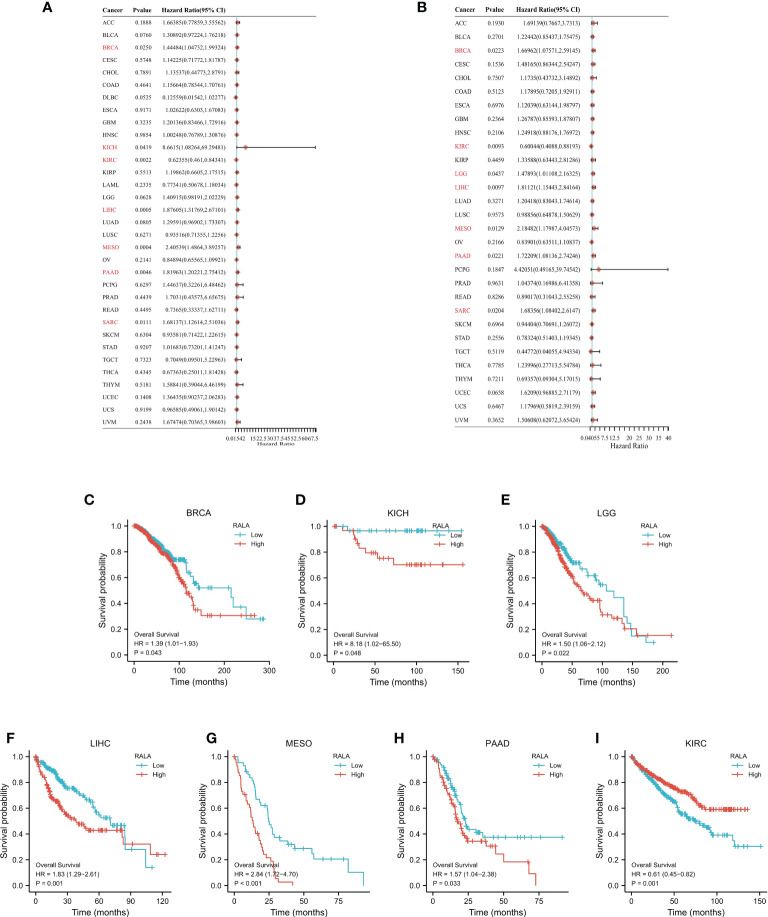
The prognostic value of RALA. The survival analysis of OS **(A)** and DSS **(B)** by RALA in pan-cancer described in the forest map. **(C–I)** K-M analysis curve of RALA expression and prognosis in different cancers.

In order to study the correlation between the expression level of RALA in pan-cancer and prognosis, we conducted survival correlation analysis on overall survival (OS) and disease special survival (DSS) of each cancer (**Figures 3A, B**). Through the analysis of cox proportional hazard model, the results showed that the expression levels of RALA were correlated with OS in breast invasive cancer (BRCA) (p=0.0250), kidney chromophobe (KICH) (p=0.0419), kidney renal clear cell cancer (KIRC) (p=0.0022), HCC (p=0.0005), MESO (p=0.0004), PAAD (p=0.0046) and Sarcoma (SARC) (p=0.0111) (**Figure 3A**); with DSS in BRCA (p=0.0223), KIRC (p=0.0093), LGG (p=0.0437), HCC (p=0.0097), MESO (p=0.0129), PAAD (p=0.0221), SARC (p=0.0204) (**Figure 3B**). The results of K-M analysis showed that the increased RALA was related to the shorter OS of BRCA, KICH, LGG, HCC, MESO and PAAD. On the contrary, in KIRC, the OS of patients with increased RALA was longer (**Figures 3C–I**; p<0.05).

### RALA is associated with immune infiltration in tumor microenvironment

**Figure 4:**
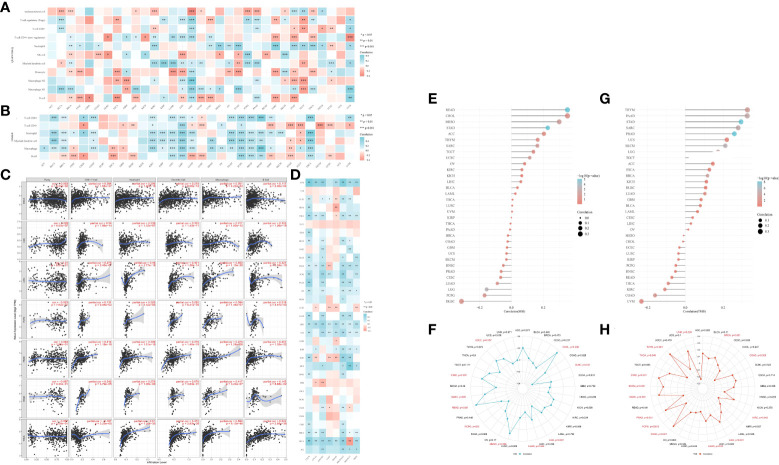
Immune infiltration of RALA in TME. **(A, B)** The heatmap of immune score and expression of RALA in multiple tumor tissues. The abscissa represents different tumor tissues, and the ordinate represents different immune score. **(C)** Correlation between RALA and tumor tissue abundance of immune infiltrates in the TIMER database. **(D)** Expression heat map of immune checkpoint related genes in pan-cancerous tissues, in which abscissa represents different immune checkpoint genes and ordinate represents different tumor tissues. **(E)** Lollipop chart and **(F)** Radar Diagram describing Spearman Correlation Analysis of MSI and RALA Expressions. **(G)** Lollipop chart and **(H)** Radar Diagram describing Spearman Correlation Analysis of TMB and RALA Expressions. *p < 0.05, **p < 0.01, ***p < 0.001.

RNA-seq data and corresponding clinical information of pan-cancer were obtained from TCGA database. For reliable immune correlation assessments, we used immunedeconv, a R package that integrates six latest algorithms, including CIBERSORT (**Supplementary Figure 2A**), EPIC (**Supplementary Figure 2B**), MCP-counter (**Supplementary Figure 2C**), QUANTISEQ (**Figure 4A**), TIMER (**Figure 4B**) and XCELL (**Supplementary Figure 2D**). The results showed that RALA was closely related to these immune cells in pan-cancer. In particular, in BRCA, LGG, HCC, PCPG, PRAD, rectum adenocarcinoma (READ), and thyroid cancer (THCA), RALA with elevated expression levels was significantly positively correlated with CD8^+^ T cell, Neutrophil, Myeloid dendritic cell, Macrophage, and B cell (**Figure 4B**), which were also validated in the TIMER database (**Figure 4C**). Up-regulation of RALA expression was significantly positively correlated with M1 macrophages in bladder urothelial cancer (BLCA), BRCA, head and neck squamous cell cancer (HNSC), LGG, HCC, LUAD, LUSC, MESO, PAAD, stomach adenocarcinoma (STAD), THCA and UVM (**Figure 4A**). On the contrary, GBM and TGCT, in which M1 macrophages were significantly negatively correlated with RALA reduction (**Figure 4A**). Then we studied the relationship between RALA expression and classical immune checkpoints, such as CD274, CTLA4, HAVCR2, LAG3, PDCD1, PDCD1LG2, SIGLEC15, TIGIT (**Figure 4D**). This suggested that RALA expression was closely associated with these common immune checkpoints in most cancers. RALA expression was closely related to the expressions of these eight immune checkpoints in BLCA and HCC, which provided a new direction for their subsequent immune-related treatment. However, there was no significant correlation between RALA and immune checkpoints analysis in cholangiocarcinoma (CHOL), DLBC, esophageal cancer (ESCA), TGCT and UCS. In addition, we found that RALA was positively correlated with microsatellite instability (MSI) in CHOL, MESO, SARC and UCEC (p<0.05), and negatively correlated with MSI in DLBC, LGG, LUAD and PCPG (**Figures 4E, F**). RALA was positively correlated with tumor mutation load (TMB) in BRCA, UCEC, LUAD and THYM (p<0.05), and negatively correlated with TMB in COAD, KIRC, THCA and UVM (**Figures 4G, H**, p<0.05).

### Mutant aspects of RALA

**Figure 5:**
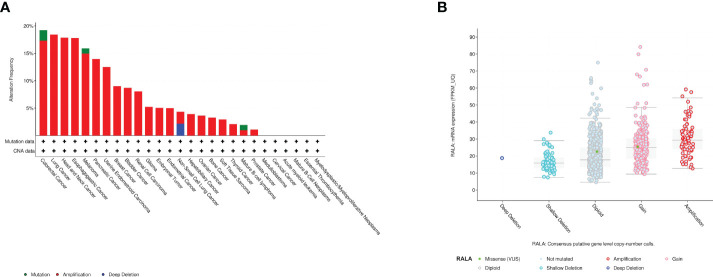
Mutant aspects of RALA. **(A)** Mutation types of RALA gene in pan-cancer. **(B)** The correlation between the mRNA obtained by RALA gene transcription in pan-cancer and the variation of tumor copy number (CNA).

By analyzing the whole genome of pan-cancer, we found that RALA has only gene amplification in most cancers, and mutations and deletions rarely appear. The main types of cancer with mutations in RALA gene are Colorectal Adenocarcinoma, Melanoma and Burkitt lymphoma. Dissimilar with other cancers, the main phenomenon of LUSC RALA gene is the deep deletion of the gene. In addition, RALA gene has almost no mutation in Follicular Thyroid Cancer, Desmoplastic/Nodular Medulloblastoma, Cervical squamous cell cancer and endocervical adenocarcinoma (CESC), LAML, Chronic Lymphocytic Leukemia/Small Lymphocytic, Essential Thrombocythemia, Follicular lymphoma, Polycythemia vera, Invasive Breast Cancer and Liposarcoma (**Figure 5A**). At the mRNA level, we analyzed the correlation between the mRNA obtained by RALA gene transcription in pan-cancer and the variation of tumor copy number alteration (CNA) (**Figure 5B**).

### Protein - protein interaction network of RALA

**Figure 6:**
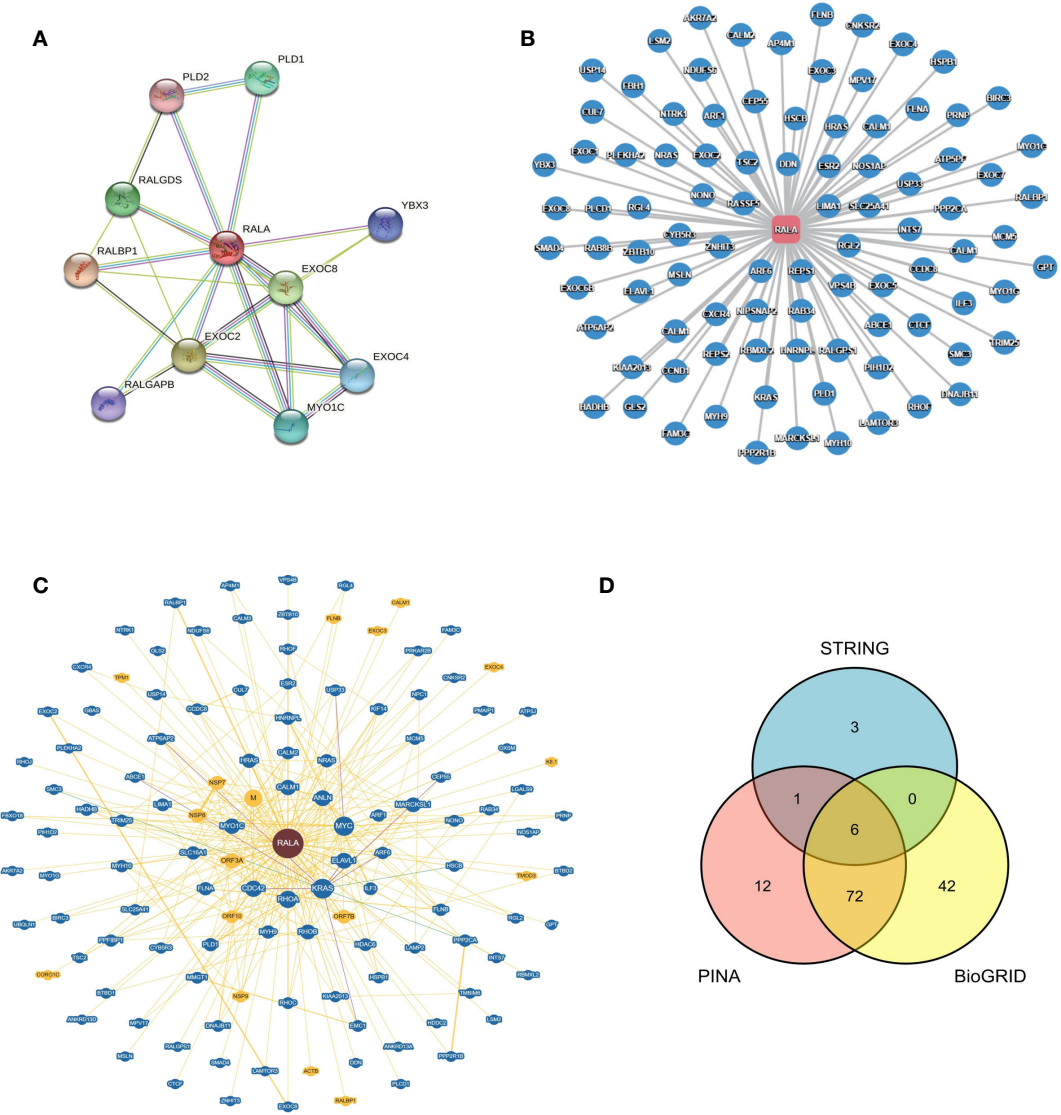
Protein - protein Interaction network of RALA in different databases. **(A)** STRING, **(B)** PINA, **(C)** BioGRID, **(D)** Venn diagram of genes interacting with RALA in three different databases.

In order to explore the protein-protein interaction network of RALA, we searched the STRING database and found 11 nodes associated with RALA, namely RALBP1, EXOC2, EXOC8, RALGDS, PLD1, MYO1C, EXOC4, YBX3, RALGAPB, PLD2 (**Figure 6A**). Except for RALGAPB, the interaction between other proteins and RALA was verified in the experiment. The database predicted that RALGAPB was related to RALA, and they were gene neighborhoods. In order to ensure the accuracy of the data, we also selected the PINA database (**Figure 6B**) and the BioGRID database (**Figure 6C**), and intersected the genes of the three databases (**Figure 6D**). The intersection genes include RALBP1, EXOC2, EXOC8, PLD1, MYO1C and EXOC4 (**Supplementary Table 1**).

### Functional analysis based on RALA expression

**Figure 7:**
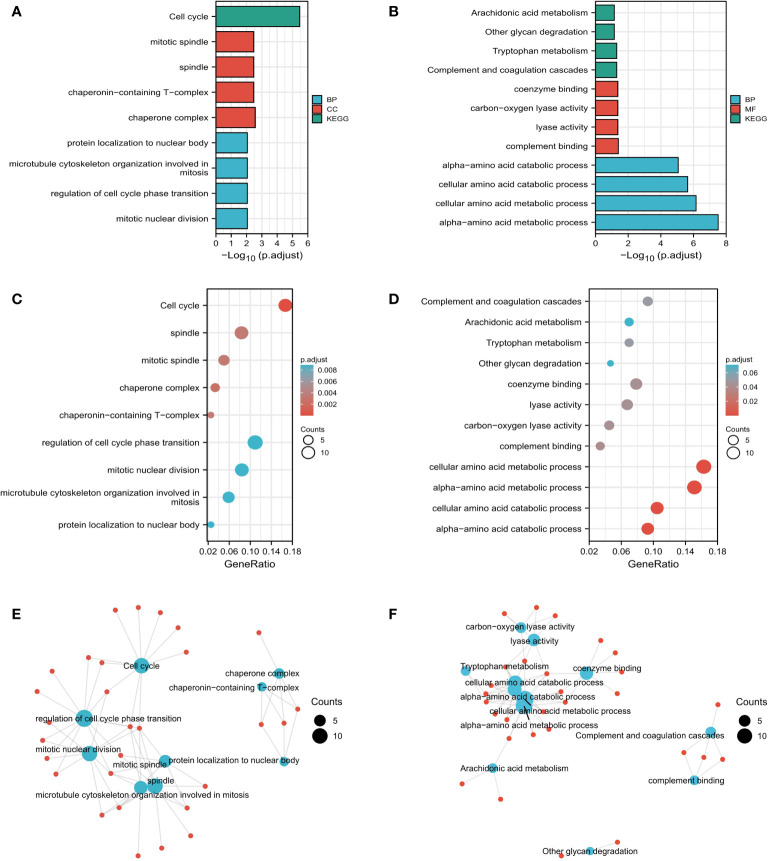
Functional analysis based on RALA expression. **(A-F)** Results of RALA functional enrichment analysis using GO and KEGG databases.

We used the pan-cancer dataset in cBioPortal to find the genes closely related to RALA and sorted them according to p-Value<0.05. We selected the genes whose absolute value of Spearman’s Correlation coefficient was more than 0.35, and screened a total of 241 genes associated with RALA (**Supplementary Tables 2**, **3**). The four molecules with the highest positive correlation with RALA were YKT6, GPSM2, GARS1 and SLC24A2 (**Supplementary Figures 3A–D**). For these genes, we used GO database and KEGG database for functional enrichment analysis (**Figures 7A–D**). Through data analysis, the expression of RALA was significantly positive correlated with mitotic nuclear division, regulation of cell cycle phase transition, microtubule cytoskeleton organization involved in mitosis and protein localization to nuclear body at the biological process (BP) level; the cellular component (CC) is associated positively with chaperone complex, chaperonin-containing T-complex, spindle and mitotic spindle; in addition, at the molecular function (MF) level, we did not enrich the function that was significantly positively correlated with RALA. Based on KEGG database, the pathways positively associated with these genes are Cell cycle (**Figures 7A, B**). For BP, RALA was negatively correlated with alpha-amino acid catabolic process, cellular amino acid catabolic process, and alpha-amino acid catabolic process. As for MF, the top four negative correlations were complement binding, lyase activity, carbon-oxygen lyase activity and coenzyme binding. No functional genes related to CC were found in RALA. In the KEGG database, the analysis results showed that the main pathways negatively correlated with RALA were complement and coagulation cascades, tryptophan metabolism, other glycan degradation and arachidonic acid metabolism (**Figures 7C–F**).

### Drugs targeting RALA

**Figure 8:**
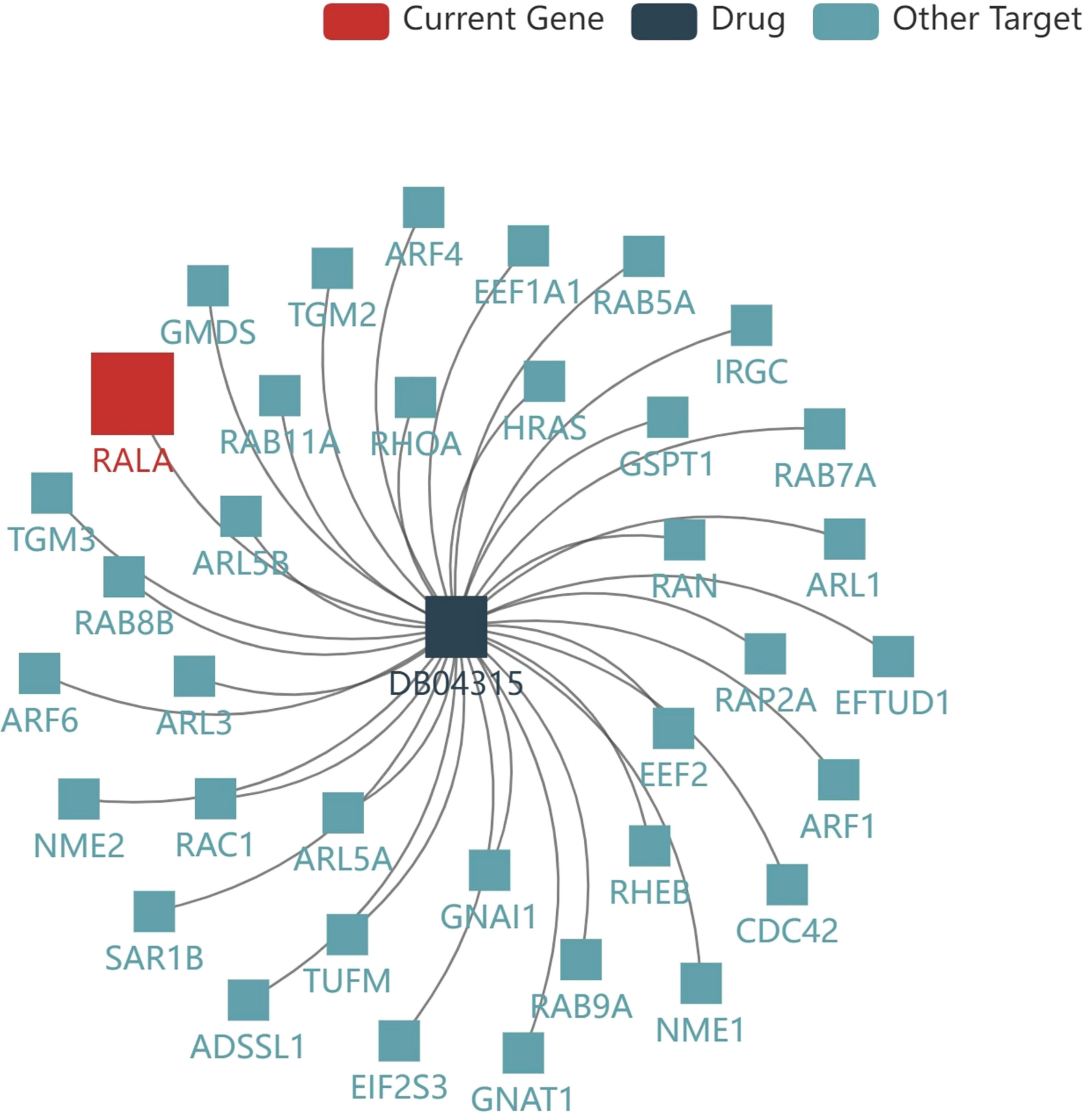
Current drug for RALA. The result showed that there was a small molecule drug targeting RALA, Guanosine-5’-Diphosphate, ID: DB04315.

TISIDB database was used to analyze the current drugs targeting RALA in DrugBank database. The result showed that there was a small molecule drug targeting RALA, Guanosine-5’-Diphosphate, ID: DB04315 (**Figure 8**). However, there is no clinical experiment to prove the relationship between the two. By reviewing the literature, we found that researchers usually focus on the clinical role of DB04315, which connects other functional groups such as diphosphate (26, 27).

### Data analysis and experimental verification of RALA in HCC

Through the above analysis, we found that the differential expression, prognosis and immune cell infiltration of RALA were statistically significant in HCC. Therefore, we chose HCC as the tumor type to conduct in-depth analysis of RALA related data.

### Expression of RALA in HCC

**Figure 9:**
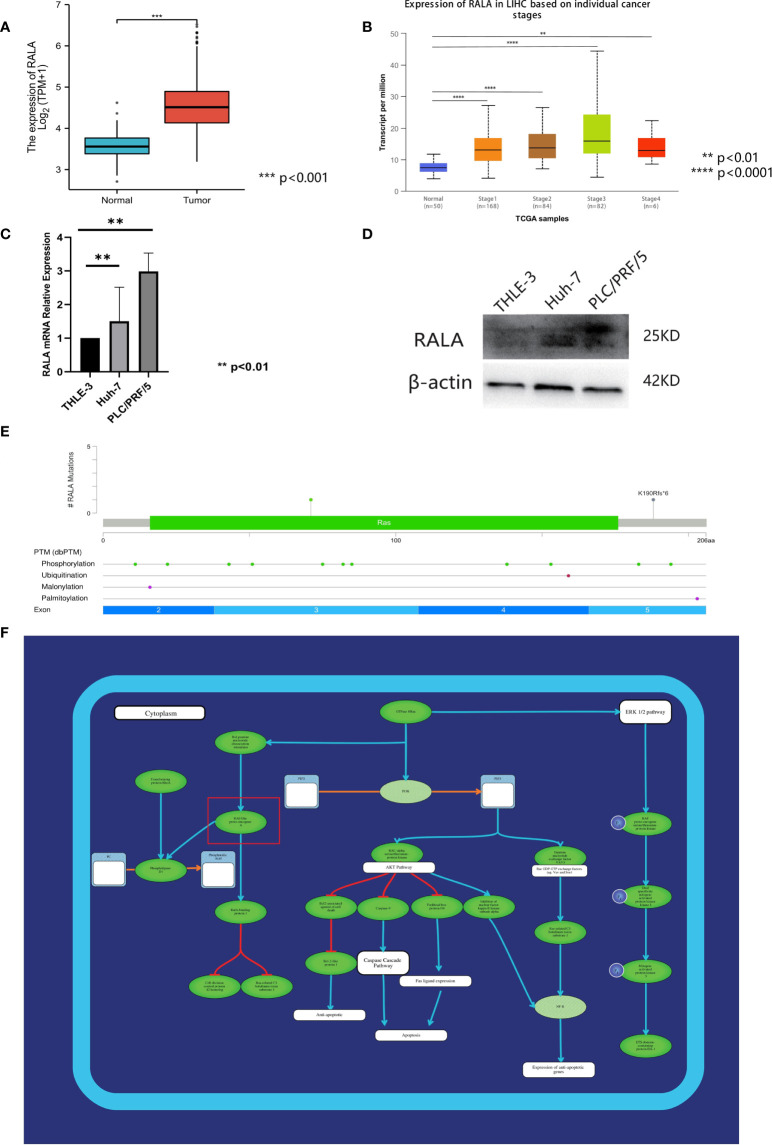
Analysis of RALA in HCC. **(A)** The expression level of RALA was predicted by TCGA database to be significantly higher in HCC tumor tissues. **(B)** The expression of RALA in different stages of HCC. **(C)** The protein expression level of RALA in HCC cell lines. **(D)** The mRNA expression level of RALA in HCC cell lines. Compared with THLE-3, the expression level of RALA was up-regulated in HCC cell lines. **(E)** Mutations and CNA data of RALA in HCC. **(F)** Signaling pathways containing RALA. **p < 0.01, ***p < 0.001, ****p < 0.0001.

The expression level of RALA was predicted by the database to be significantly higher in HCC tumor tissues than in normal tissues (**Figure 9A**) and varied by stage (**Figure 9B**). The expression of RALA in I/II/III stage were significantly higher than that in normal tissues. In addition, the expression of RALA gradually increased with the progression of the disease. However, we found that the expression level of RALA decreased in IV stage, which was similar to that in stage 1, but still higher than that in normal tissues.

### Expression of RALA in human HCC cell lines

In this study, the expression levels of RALA in HCC cell lines Huh-7, PLC/PRF/5 and liver immortalized cell line THLE-3 were analyzed. RT-qPCR results showed that compared with THLE-3, the expression level of RALA was up-regulated in HCC cell lines (**Figure 9C**). The results of Western Blotting analysis were consistent with those of RT-qPCR (**Figure 9D**).

### Mutation of RALA in HCC

We used 366 samples of HCC with mutations and CNA data in the TCGA database, of which the more significant mutation type was missense mutation (**Figure 9E**). At the gene level, the mutation site was located in the third region of the exon. At the protein level, there are also some changes in post-translational modification after gene mutation. There are 11 phosphorylation sites, 1 ubiquitination site, 1 malonylation site, and 1 palmitoylation site. These mutation sites are evenly distributed in each part of the exon, exon 2 has two phosphorylation sites, one malonylation site, exon 3 includes five phosphorylation sites, exon 4 contains two phosphorylation sites, one ubiquitination site.

### The correlation between RALA and HCC signaling pathways

**Figure 10:**
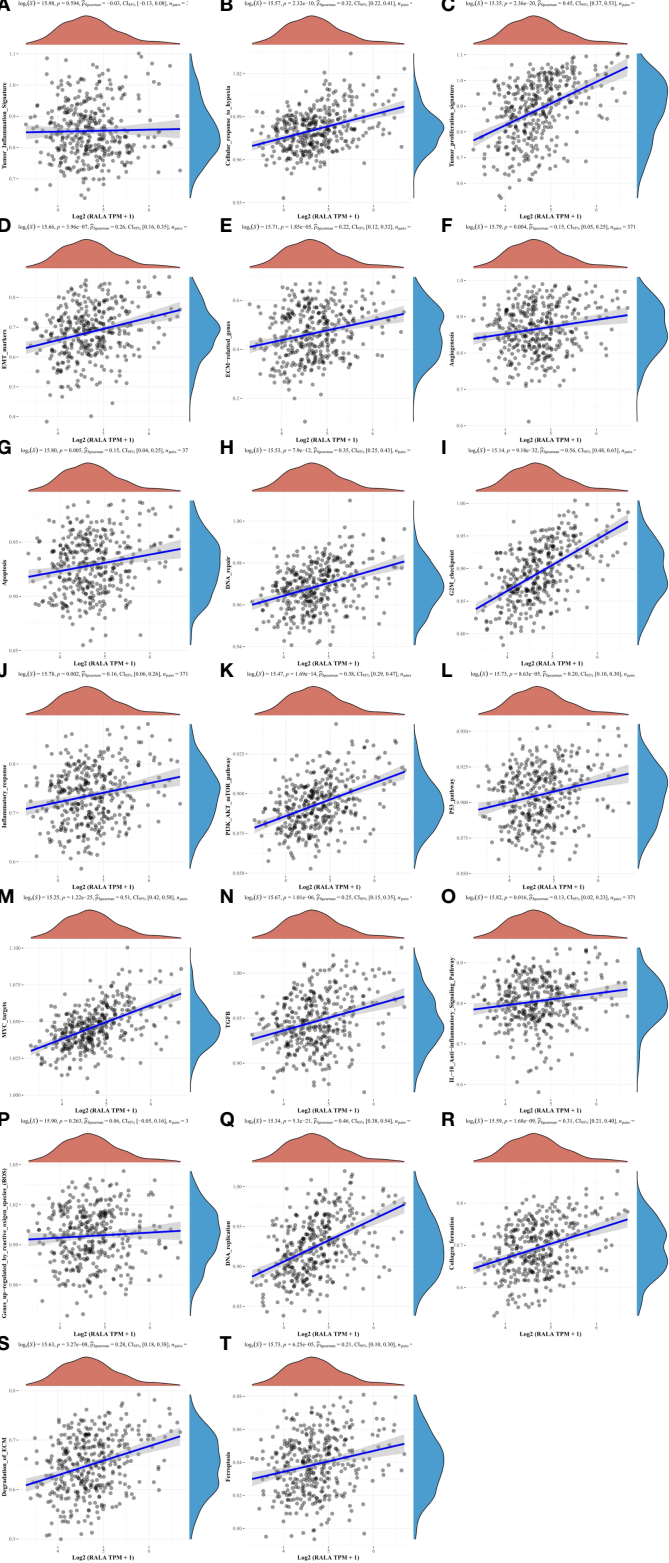
The correlation between RALA and HCC signaling pathways. **(A, P)** There is no significant correlation between RALA and tumor inflammation signature, genes up-regulated by reactive oxygen species. **(B–O, Q–T)** RALA is remarkable positively correlated with cellular response to hypoxia, angiogenesis and other common intracellular signaling pathways.

RAS signaling pathway is one of the main channels to govern cell proliferation, survival and apoptosis inhibiting procedures in response to intracellular signals transduced by mitogens. RALA is a downstream molecule of RAS signaling pathway (**Figure 9F**). Next, we use HCC data in TCGA to further analyze the correlation between RALA and gene pathway. The results showed that the pathways significantly associated with RALA were mainly cellular response to hypoxia, tumor proliferation signature, EMT markers, ECM related genes, angiogenesis, apoptosis, DNA repair, G2M checkpoint, Inflammatory response. PI3K-AKT-mTOR pathway, P53 pathway, MYC targets, TGFB, IL-10 anti-inflammatory signaling pathway, DNA replication, collagen formation, degradation of ECM and ferroptosis, and the relationship between RALA and these pathways was positively correlated (**Figure 10**).

## Discussion

The results of data analysis showed that RALA gene was highly expressed in 29 kinds of cancers. The expression level of RALA in normal tissues was higher than that in cancer tissues only in LAML, LUAD, LUSC and PCPG. IHC results also supported the conclusion. We used TISIDB database to analyze the relationship between RALA expression and tumor staging in pan-cancer. The data showed that the staging of colorectal cancer was not significantly correlated with the expression of RALA. The study of Ushigome M et al. also proved this conclusion. RALA protein as a tumor antigen can induce the production of serum RALA antibody (s-RALA-Abs). They analyzed 314 patients with colorectal cancer and found that the level of RALA expression in 0/I/II stage of cancer was similar to that in III/IV stage. However, the recurrence-free survival rate in the s-RALA-Abs positive group was significantly poor (28).

RALA and RALB are small GTPases related to the growth and metastasis of various cancers. Although they belong to the RAS superfamily of small G proteins and are highly homologous small G proteins, their roles in BRCA are completely opposite. Thies KA et al. have shown that RALA knockdown inhibits *in situ* tumor growth in TNBC cells, while RALB knockdown accelerates this process. For the metastatic ability of tumor, similar results were obtained. RALA promoted the metastatic growth of TNBC cell lines, while RALB inhibited tumor metastasis (29). This experiment emphasized the potential of targeted RALA in the treatment of BRCA. But in bladder cancer, researchers found different phenomena. Oxford et al. found elevated levels of GTP-RALA in bladder cancer cell lines UMUC-3 and DU145, but in the experimental results obtained by transwell, only RALB was necessary for cancer cell migration and RALA inhibited it (30). In the early studies of multiple myeloma (MM), experiments had shown that RALB can promote the migration of OPM-1 and NCI-H929 cells, but RALA did not have this effect (31). In HCC, the expression level of RALA and the level of RALA autoantibodies in cancer tissues were significantly higher than those in patients with liver cirrhosis or normal tissues. RALA knockout greatly reduced the proliferation and invasion ability of cells *in vitro* (32). In addition, RALA silencing also reduced the stemness of HCC cells, and over-expression of RALA could reverse these characteristics, while RALB was not found to function in HCC tissues (14). Therefore, some researchers believed that RALA rather than RALB can be used as a therapeutic target for HCC.

We also analyzed the interaction between RALA and tumor immunity. TME is rich in immune cell infiltration, such as tumor associated macrophages (TAM), neutrophils, antigen presenting cells (APC), and adaptive immune cells. It is of great significance for the therapeutic effect and prognosis of cancer patients, and can be used as a marker to evaluate the response of tumor cells to immunotherapy (33). RALA GTPases can also be activated in NK cells and mediate cytotoxicity. After silencing both RALA and RALB, the cytotoxicity of NK cells is evidently attenuated (12). The purpose of immunotherapy is to enhance the body’s natural defense ability to eliminate malignant cells. It is a major breakthrough in cancer treatment. In recent years, great progress has been made in basic and clinical research, and immune cells are the cellular basis of tumor immunotherapy (34). According to the TCGA datasets, we conclude that RALA is significantly associated with multiple immune cells in pan-cancer. Cole G et al. used DNA vaccine technology to inject RALA probe into mice with prostate cancer by microneedle delivery system, and used vaccine to induce tumor-specific cellular immune response, so as to achieve the purpose of cancer treatment (35). Similar studies had been conducted in cervical cancer (36, 37). Ali AA et al. designed MN/RALA-E6/E7-vaccine using a new system for delivering therapeutic HPV DNA vaccines. The vaccine had a significantly preventive effect on cervical cancer and can significantly reduce tumor mass and prolong survival (36). RALA is a tumor antigen, so s-RALA-Abs could be used as potential biomarkers. This study had been reported in HCC, ESCA, colorectal cancer (38), breast cancer, ovarian cancer (39) and gastric cancer (40). Nanami T, et al. found that in gastric cancer, the presence of s-RALA-Abs had nothing to do with other conventional serum tumor markers. In clinical tests, the detection rate of gastric cancer can be improved when s-RALA-Abs is combined with CEA and CA19-9 (40). By detecting s-RALA-Abs and s-p53-Abs in 1833 patients with different cancers, it was found that the positive rates of both antibodies were significantly increased in all types of cancers. Therefore, the combination of s-RALA-Abs and s-p53-Abs has a synergistic effect on the diagnosis of cancer (41). RALA also inhibits cancer cell migration in certain types of tumors, such as bladder cancer. Interestingly, RALA expression was up-regulated in tumor tissues of bladder cancer by analysis of the TCGA database (30). Similarly, RALA expression was down-regulated in tumor tissues of LUSC and LUAD, but researchers found that inhibition of the RALA signaling pathway could treat non-small cell lung cancer (42). We did not find any studies on the expression and function of RALA in PCPG. In addition, we also found that RALA was significantly associated with several common immune checkpoints in pan-cancer, and the expression of RALA was closely related to the biological process of most immune-related molecules. However, there are few clinical studies on RALA and immune checkpoint inhibitors in pan-cancer. Our data analysis can provide new ideas for cancer treatment and new targets for the development of immune inhibitors.

Through the enrichment analysis of RALA gene, the results showed that RALA high expression may be through mitotic nuclear division, regulation of cell cycle phase transition, microtubule cytoskeleton organization involved in mitotic, protein localization to nuclear body. Chaperone complex, chaperonin-containing T-complex, spindle, mitotic spindle and cell cycle these biological processes affect the progression of cancer. These results are consistent with previously confirmed studies suggesting that RALA signaling regulates cell proliferation and migration (43).

Overall, our extensive cancer research on RALA showed significant differential expression of this gene between tumor and normal tissues, and elucidated the correlation between RALA expression and immune cell infiltration and common immune checkpoints (44). RALA affects the prognosis of a variety of tumors through extensive data analysis (45). For pan-cancer, the level of its expression might be closely related to tumor staging, and the specific function of RALA in each cancer needs to be further studied. Furthermore, the relationship between RALA expression and TMB, MSI was statistically significant in a variety of cancers. Through data analysis, we enriched the molecular functions and signaling pathways of RALA. In summary, our findings may help explain the role of RALA in the development and progression of pan-cancer (46).

## Conclusion

In combination with the pan-cancer cohort, we analyzed multiple databases and found that the up-regulation of RALA expression was related to poor prognosis of tumors, which might be involved in the signaling pathway related to immune cell infiltration. Since RALA was significantly associated with a variety of immune checkpoints, it could be used as a potential target for immunotherapy. However, further clinical and general biological studies are needed to verify the function of RALA in tumor cells.

## Data availability statement

The original contributions presented in the study are included in the article/**Supplementary Material**. Further inquiries can be directed to the corresponding authors.

## Author contributions

LZ designed the study. HJ and LZ drafted the manuscript. HJ, SQ, JH, JX, QL, YM and LZ collected the data and conducted the picture processing. LZ and YM revised the manuscript. All authors have read and approved the final version of manuscript.

## Funding

This study was supported by the National Natural Science Foundation of China (81602575, 81701847, 61572200), Natural Science Foundation of Hunan Province (2022JJ30794), Xiang Jiang Scholars Program/Hong Kong Scholars Program (XJ2016054), Basic Research Project of Changsha Science and Technology Plan (kq2004126), Changsha Municipal Natural Science Foundation (kq2202126), Graduate Education and Teaching Reform Project of Central South University (2022YJSKS018), the Student Innovation Project of Central South University (2022ZZTS0267), the Postgraduate Scientific Research Innovation Project of Hunan Province (CX20220349).

## Conflict of interest

The authors declare that the research was conducted in the absence of any commercial or financial relationships that could be construed as a potential conflict of interest.

## Publisher’s note

All claims expressed in this article are solely those of the authors and do not necessarily represent those of their affiliated organizations, or those of the publisher, the editors and the reviewers. Any product that may be evaluated in this article, or claim that may be made by its manufacturer, is not guaranteed or endorsed by the publisher.

## Supplementary material

Supplementary Figure 1 RALA expression analyzed by TCGA and GETx datasets. **(A)** The results showed that RALA expression was higher in cancer tissues than in normal tissues in most tumors. **(B)** The mRNA expression of RALA in normal tissues.

Supplementary Figure 2 Immune infiltration of RALA in TME. We use four latest algorithms: **(A)** CIBERSORT, **(B)** EPIC, **(C)** MCP-counter and **(D)** xCell. *p < 0.05, **p < 0.01, ***p < 0.001.

Supplementary Figure 3 The four molecules with the highest positive correlation with RALA. **(A)** YKT6, **(B)** GPSM2, **(C)** GARS1, **(D)** SLC24A2.

Supplementary Table 1 Genes that interact with RALA in three different databases.

Supplementary Table 2 129 genes co-expressed with RALA in pan-cancer. Their Spearman ‘s correlation coefficient > 0.35.

Supplementary Table 3 112 genes co-expressed with RALA in pan-cancer. Their Spearman ‘s correlation coefficient < -0.35.

## Glossary

**Table:**

ACC	adrenocortical carcinoma
APC	antigen presenting cells
BP	biological pathway
BRCA	breast invasive carcinoma
BLCA	bladder urothelial carcinoma
CC	cellular component
CESC	Cervical squamous cell carcinoma and endocervical adenocarcinoma
CHOL	cholangiocarcinoma
CNA	copy number alteration
COADREAD	Colon adenocarcinoma/Rectum adenocarcinoma Esophageal carcinoma
TSCs	tumor stem cells
CTLA-4	cytotoxic T lymphocyte-associated antigen 4;
DLBC	lymphoid neoplasm diffuse large B-cell lymphoma
DSS	disease special survival
ESCA	esophageal carcinoma
GETx	Genotype-Tissue Expression
GO	Gene Ontology
HCC	hepatocellular carcinoma
HNSC	head and neck squamous cell carcinoma
HPA	Human Protein Atlas
ICIs	lmmune checkpoint inhibitors
IF	immunofluorescence
IHC	Immunohistochemistry
irAEs	immune-related adverse events
KICH	kidney chromophobe
KIRC	kidney renal clear cell carcinoma
LAML	acute myeloid leukemia
LGG	low grade glioma
LIHC	liver hepatocellular carcinoma
LUAD	lung adenocarcinoma
MESO	mesothelioma
MF	molecular function
MM	multiple myeloma
MSI	microsatellite instability;
NK cells	natural killer cells
nTPM	normalized transcript per million
OS	overall survival
OV	ovarian serous cystadenocarcinoma
PAAD	pancreatic adenocarcinoma
PCPG	pheochromocytoma and paraganglioma
PD-1	programmed cell death 1
PD-L1	programmed cell death ligand 1
Raslike	Ral
READ	rectum adenocarcinoma
SDS-PAGE	sodium dodecyl sulfate polyacrylamide gelelectrophoresis
STAD	stomach adenocarcinoma
TAM	tumor associated macrophages
TCGA	the Cancer Genome Atlas
TGCT	testicular cancer
THCA	thyroid carcinoma
TMB	tumor mutational burden
TME	tumor microenvironment
TPM	transcripts per 100 000 reads
UCEC	uterine corpus endometrial carcinoma
UCS	uterine carcinosarcoma
UVM	ocular melanomas

## References

[1] Sung H Ferlay J Siegel RL Laversanne M Soerjomataram I Jemal A. Global cancer statistics 2020: GLOBOCAN estimates of incidence and mortality worldwide for 36 cancers in 185 countries. CA Cancer J Clin (2021) 71(3):209–49. doi: 10.3322/caac.21660 33538338

[2] Zhang H Dai Z Wu W Wang Z Zhang N Zhang L. Regulatory mechanisms of immune checkpoints PD-L1 and CTLA-4 in cancer. J Exp Clin Cancer Res (2021) 40(1):184. doi: 10.1186/s13046-021-01987-7 34088360 PMC8178863

[3] Ramos-Casals M Brahmer JR Callahan MK Flores-Chavez A Keegan N Khamashta MA. Immune-related adverse events of checkpoint inhibitors. Nat Rev Dis Primers (2020) 6(1):38. doi: 10.1038/s41572-020-0160-6 32382051 PMC9728094

[4] Sun Y. Translational horizons in the tumor microenvironment: harnessing breakthroughs and targeting cures. Med Res Rev (2015) 35(2):408–36. doi: 10.1002/med.21338 PMC4374701 25588753

[5] Yahya EB Alqadhi AM. Recent trends in cancer therapy: A review on the current state of gene delivery. Life Sci (2021) 269:119087. doi: 10.1016/j.lfs.2021.119087 33476633

[6] Lee YT Tan YJ Oon CE. Molecular targeted therapy: Treating cancer with specificity. Eur J Pharmacol (2018) 834:188–96. doi: 10.1016/j.ejphar.2018.07.034 30031797

[7] Sun W Shi Q Zhang H Yang K Ke Y Wang Y. Advances in the techniques and methodologies of cancer gene therapy. Discov Med (2019) 27(146):45–55. 30721651

[8] van Dam EM Robinson PJ. Ral: mediator of membrane trafficking. Int J Biochem Cell Biol (2006) 38(11):1841–7. doi: 10.1016/j.biocel.2006.04.006 16781882

[9] Bos JL. All in the family? new insights and questions regarding interconnectivity of ras, Rap1 and ral. EMBO J (1998) 17(23):6776–82. doi: 10.1093/emboj/17.23.6776 PMC1171024 9843482

[10] Tian X Rusanescu G Hou W Schaffhausen B Feig LA. PDK1 mediates growth factor-induced ral-GEF activation by a kinase-independent mechanism. EMBO J (2002) 21(6):1327–38. doi: 10.1093/emboj/21.6.1327 PMC125928 11889038

[11] Moghadam AR Patrad E Tafsiri E Peng W Fangman B Pluard TJ. Ral signaling pathway in health and cancer. Cancer Med (2017) 6(12):2998–3013. doi: 10.1002/cam4.1105 29047224 PMC5727330

[12] Sanchez-Ruiz J Mejias R Garcia-Belando M Barber DF Gonzalez-Garcia A. Ral GTPases regulate cell-mediated cytotoxicity in NK cells. J Immunol (2011) 187(5):2433–41. doi: 10.4049/jimmunol.1003089 21810610

[13] Urano T Emkey R Feig LA. Ral-GTPases mediate a distinct downstream signaling pathway from ras that facilitates cellular transformation. EMBO J (1996) 15(4):810–6. doi: 10.1002/j.1460-2075.1996.tb00416.x PMC450279 8631302

[14] Tian L Zhao L Sze KM Kam CS Ming VS Wang X. Dysregulation of RalA signaling through dual regulatory mechanisms exerts its oncogenic functions in hepatocellular carcinoma. Hepatology (2021) 76(1):48–65. doi. doi: 10.1002/hep.32236 PMC9299834 34767674

[15] Addeo A Friedlaender A Banna GL Weiss GJ. TMB or not TMB as a biomarker: That is the question. Crit Rev Oncol Hematol (2021) 163:103374. doi: 10.1016/j.critrevonc.2021.103374 34087341

[16] Snyder A Makarov V Merghoub T Yuan J Zaretsky JM Desrichard A. Genetic basis for clinical response to CTLA-4 blockade in melanoma. N Engl J Med (2014) 371(23):2189–99. doi: 10.1056/NEJMoa1406498 PMC4315319 25409260

[17] Rizzo A Ricci AD Brandi G. PD-L1, TMB, MSI, and other predictors of response to immune checkpoint inhibitors in biliary tract cancer. Cancers (Basel) (2021) 13(3):558. doi: 10.3390/cancers13030558 33535621 PMC7867133

[18] Thorsson V Gibbs DL Brown SD Wolf D Bortone DS Ou Yang TH. The immune landscape of cancer. Immunity (2018) 48(4):812–830.e814. doi: 10.1016/j.immuni.2018.03.023 29628290 PMC5982584

[19] Bonneville R Krook MA Kautto EA Miya J Wing MR Chen HZ. Landscape of microsatellite instability across 39 cancer types. JCO Precis Oncol (2017) 2017:PO.17.000735. doi: 10.1200/PO.17.00073 29850653 PMC5972025

[20] Gao J Aksoy BA Dogrusoz U Dresdner G Gross B Sumer SO. Integrative analysis of complex cancer genomics and clinical profiles using the cBioPortal. Sci Signal (2013) 6(269):pl1. doi: 10.1126/scisignal.2004088 23550210 PMC4160307

[21] Szklarczyk D Gable AL Lyon D Junge A Wyder S Huerta-Cepas J. STRING v11: protein-protein association networks with increased coverage, supporting functional discovery in genome-wide experimental datasets. Nucleic Acids Res (2019) 47(D1):D607–13. doi: 10.1093/nar/gky1131 PMC6323986 30476243

[22] Frolkis A Knox C Lim E Jewison T Law V Hau DD. SMPDB: The small molecule pathway database. Nucleic Acids Res (2010) 38(Database issue):D480–7. doi: 10.1093/nar/gkp1002 PMC2808928 19948758

[23] Jewison T Su Y Disfany FM Liang Y Knox C Maciejewski A. SMPDB 2.0: big improvements to the small molecule pathway database. Nucleic Acids Res (2014) 42(Database issue):D478–84. doi: 10.1093/nar/gkt1067 PMC3965088 24203708

[24] Hanzelmann S Castelo R Guinney J. GSVA: gene set variation analysis for microarray and RNA-seq data. BMC Bioinf (2013) 14:7. doi: 10.1186/1471-2105-14-7 PMC3618321 23323831

[25] Wei J Huang K Chen Z Hu M Bai Y Lin S. Characterization of glycolysis-associated molecules in the tumor microenvironment revealed by pan-cancer tissues and lung cancer single cell data. Cancers (Basel) (2020) 12(7):1788. doi: 10.3390/cancers12071788 32635458 PMC7408567

[26] Rasouly A Pani B Nudler E. A magic spot in genome maintenance. Trends Genet (2017) 33(1):58–67. doi: 10.1016/j.tig.2016.11.002 27931778 PMC5182163

[27] Wan L Zhu Y Li W Zhang W Mu W. Combinatorial modular pathway engineering for guanosine 5'-diphosphate-l-fucose production in recombinant escherichia coli. J Agric Food Chem (2020) 68(20):5668–75. doi: 10.1021/acs.jafc.0c01064 32336091

[28] Ushigome M Shimada H Nabeya Y Shiratori F Soda H Takiguchi N. Possible predictive significance of serum RalA autoantibodies on relapse-free survival in patients with colorectal cancer. Mol Clin Oncol (2021) 14(1):18. doi: 10.3892/mco.2020.2180 33363728 PMC7725215

[29] Thies KA Cole MW Schafer RE Spehar JM Richardson DS Steck SA. The small G-protein RalA promotes progression and metastasis of triple-negative breast cancer. Breast Cancer Res (2021) 23(1):65. doi: 10.1186/s13058-021-01438-3 34118960 PMC8196523

[30] Oxford G Owens CR Titus BJ Foreman TL Herlevsen MC Smith SC. RalA and RalB: antagonistic relatives in cancer cell migration. Cancer Res (2005) 65(16):7111–20. doi: 10.1158/0008-5472.CAN-04-1957 16103060

[31] de Gorter DJ Reijmers RM Beuling EA Naber HP Kuil A Kersten MJ. The small GTPase ral mediates SDF-1-induced migration of b cells and multiple myeloma cells. Blood (2008) 111(7):3364–72. doi: 10.1182/blood-2007-08-106583 18227351

[32] Ezzeldin M Borrego-Diaz E Taha M Esfandyari T Wise AL Peng W. RalA signaling pathway as a therapeutic target in hepatocellular carcinoma (HCC). Mol Oncol (2014) 8(5):1043–53. doi: 10.1016/j.molonc.2014.03.020 PMC5528517 24785097

[33] Bejarano L Jordao MJC Joyce JA. Therapeutic targeting of the tumor microenvironment. Cancer Discovery (2021) 11(4):933–59. doi: 10.1158/2159-8290.CD-20-1808 33811125

[34] Zhang Y Zhang Z. The history and advances in cancer immunotherapy: understanding the characteristics of tumor-infiltrating immune cells and their therapeutic implications. Cell Mol Immunol (2020) 17(8):807–21. doi: 10.1038/s41423-020-0488-6 PMC7395159 32612154

[35] Cole G Ali AA McErlean E Mulholland EJ Short A McCrudden CM. DNA Vaccination via RALA nanoparticles in a microneedle delivery system induces a potent immune response against the endogenous prostate cancer stem cell antigen. Acta Biomater (2019) 96:480–90. doi: 10.1016/j.actbio.2019.07.003 31299353

[36] Ali AA McCrudden CM McCaffrey J McBride JW Cole G Dunne NJ. DNA Vaccination for cervical cancer; a novel technology platform of RALA mediated gene delivery via polymeric microneedles. Nanomedicine (2017) 13(3):921–32. doi: 10.1016/j.nano.2016.11.019 27979747

[37] Cole G Ali AA McCrudden CM McBride JW McCaffrey J Robson T. DNA Vaccination for cervical cancer: Strategic optimisation of RALA mediated gene delivery from a biodegradable microneedle system. Eur J Pharm Biopharm (2018) 127:288–97. doi: 10.1016/j.ejpb.2018.02.029 29510205

[38] Ushigome M Nabeya Y Soda H Takiguchi N Kuwajima A Tagawa M. Multi-panel assay of serum autoantibodies in colorectal cancer. Int J Clin Oncol (2018) 23(5):917–23. doi: 10.1007/s10147-018-1278-3 29691673

[39] Sun H Shi JX Zhang HF Xing MT Li P Dai LP. Serum autoantibodies against a panel of 15 tumor-associated antigens in the detection of ovarian cancer. Tumour Biol (2017) 39(6):1010428317699132. doi: 10.1177/1010428317699132 28618923

[40] Nanami T Hoshino I Ito M Yajima S Oshima Y Suzuki T. Prevalence of autoantibodies against ras-like GTPases, RalA, in patients with gastric cancer. Mol Clin Oncol (2020) 13(4):28. doi: 10.3892/mco.2020.2098 32765875 PMC7403843

[41] Nanami T Hoshino I Shiratori F Yajima S Oshima Y Suzuki T. Presence of serum RalA and serum p53 autoantibodies in 1833 patients with various types of cancers. Int J Clin Oncol (2022) 27(1):72–6. doi: 10.1007/s10147-021-02045-0 34632560

[42] Male H Patel V Jacob MA Borrego-Diaz E Wang K Young DA. Inhibition of RalA signaling pathway in treatment of non-small cell lung cancer. Lung Cancer (2012) 77(2):252–9. doi: 10.1016/j.lungcan.2012.03.007 22498113

[43] Cemeli T Guasch-Valles M Ribes-Santolaria M Ibars E Navaridas R Dolcet X. Antitumor effects of ral-GTPases downregulation in glioblastoma. Int J Mol Sci (2022) 23(15):8199. doi: 10.3390/ijms23158199 35897776 PMC9330696

[44] Wang P Zhang W Wang L Liang W Cai A Gao Y. RCC2 interacts with small GTPase RalA and regulates cell proliferation and motility in gastric cancer. Onco Targets Ther (2020) 13:3093–103. doi: 10.2147/OTT.S228914 PMC7166089 32341655

[45] Gong S Chen Y Meng F Zhang Y Wu H Li C. RCC2, a regulator of the RalA signaling pathway, is identified as a novel therapeutic target in cisplatin-resistant ovarian cancer. FASEB J (2019) 33(4):5350–65. doi: 10.1096/fj.201801529RR 30768358

[46] Yan C Theodorescu D. RAL GTPases: Biology and potential as therapeutic targets in cancer. Pharmacol Rev (2018) 70(1):1–11. doi: 10.1124/pr.117.014415 29196555 PMC5712631

