# Supplementary figures and images for: Systematic pan-cancer analysis identifies RALA as a tumor targeting immune therapeutic and prognostic marker
Source: Front Immunol. 2022 Nov 17;13:1046044. doi: 10.3389/fimmu.2022.1046044 (PMC9713825; doi:10.3389/fimmu.2022.1046044)

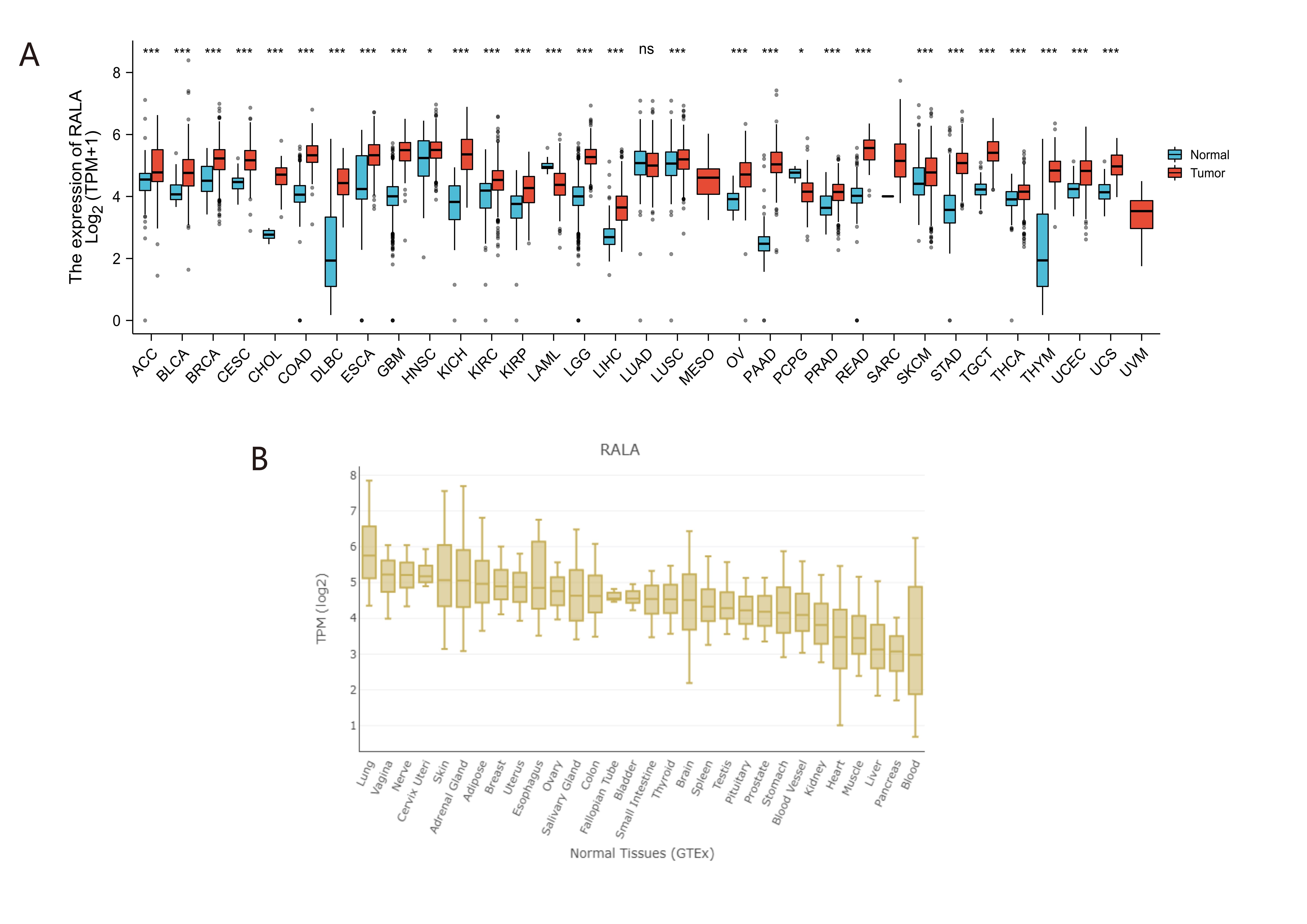

Supplement: Supplementary Figure 1 — RALA expression analyzed by TCGA and GETx datasets. (A) The results showed that RALA expression was higher in cancer tissues than in normal tissues in most tumors. (B) The mRNA expression of RALA in normal tissues. [file Image_1.jpeg]

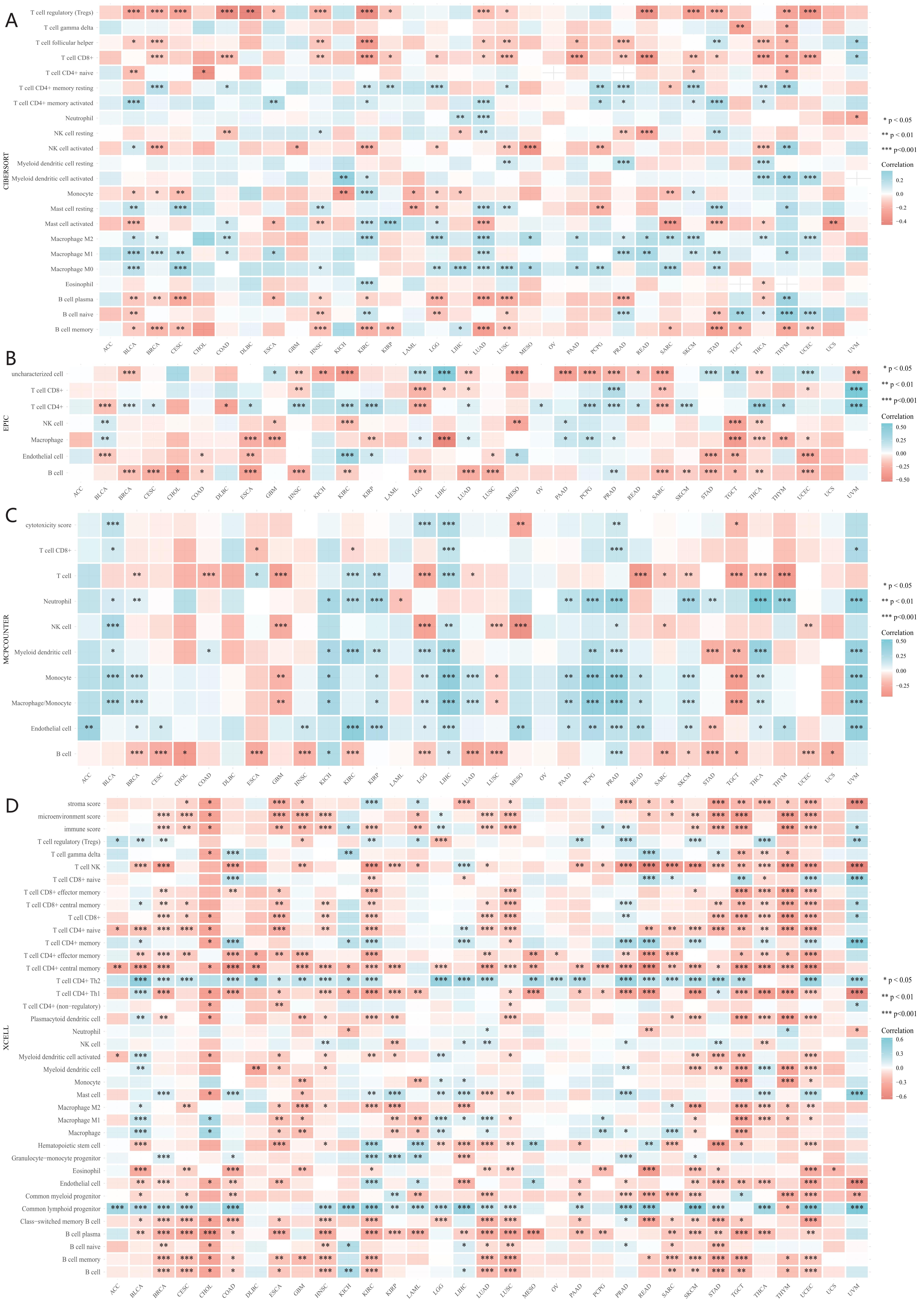

Supplement: Supplementary Figure 2 — Immune infiltration of RALA in TME. We use four latest algorithms: (A) CIBERSORT, (B) EPIC, (C) MCP-counter and (D) xCell. *p < 0.05, **p < 0.01, ***p < 0.001. [file Image_2.jpeg]

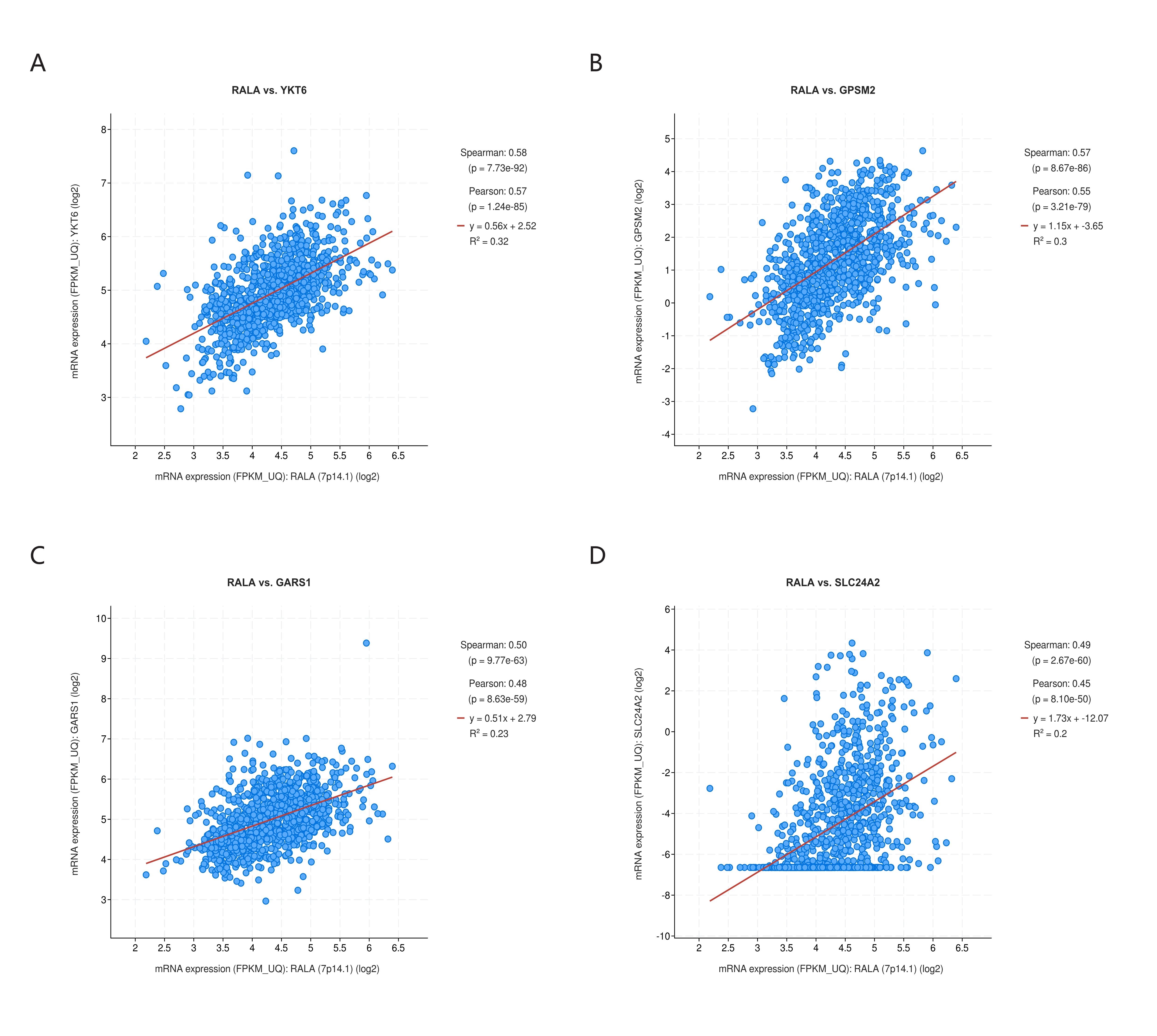

Supplement: Supplementary Figure 3 — The four molecules with the highest positive correlation with RALA. (A) YKT6, (B) GPSM2, (C) GARS1, (D) SLC24A2. [file Image_3.jpeg]
